# Omics-Guided Insights into Nanoparticle Complexity and Neural Regeneration

**DOI:** 10.3390/bios16050239

**Published:** 2026-04-24

**Authors:** Yujung Chang, Sungwoo Lee, Garam Yang, Seung Seon Yang, Min Park, Jessica Kim, Yoon Ha, Sungho Park, Junsang Yoo

**Affiliations:** 1 Laboratory of Regenerative Medicine for Neurodegenerative Disease, Stand Up Therapeutics Inc., Hannamdaero 98, Seoul 04418, Republic of Korea; drchang@stutps.com (Y.C.); rivership@kaist.ac.kr (G.Y.); seungseon@stutps.com (S.S.Y.); jkim-27@peddie.org (J.K.); 2 Department of Chemistry, Yonsei University, Seoul 03722, Republic of Korea; sungwoo22@skku.edu; 3 Department of Biological Sciences, Korea Advanced Institute of Science and Technology, Daejeon 34141, Republic of Korea; 4 Department of Chemical Engineering and Biochemistry, Northeastern University, Boston, MA 02115, USA; park.min2@northeastern.edu; 5 Spine & Spinal Cord Center, Department of Neurosurgery, College of Medicine, Yonsei University, Seoul 03722, Republic of Korea

**Keywords:** plasmonic nanoparticles, neuronal regeneration, omics-guided engineering, cellular aging, nanoparticle complexity

## Abstract

Structurally complex plasmonic nanoarchitectures represent an emerging class of nanomaterials with properties that extend beyond those of conventional spherical nanoparticles. Their distinctive structural motifs generate dense near field electromagnetic hot spots, expand interfacial surface area, and create biophysical environments at the nano–bio interface that can actively engage cellular signaling networks relevant to neural regeneration and aging. Despite growing interest in these platforms, a systematic, omics-guided synthesis that links nanoparticle structural features to transcriptomic programs and regenerative outcomes has been lacking. In this review, we summarize recent advances in high complexity plasmonic nanoparticle engineering and integrate published omics-based evidence of their cellular effects, organizing the discussion. Across these studies, transcriptomic analyses of nanoparticle treated neural systems consistently highlight three convergent biological themes: mitigation of oxidative stress and activation of antioxidant pathways, suppression of neuroinflammatory signaling, and induction of neuronal developmental and plasticity programs. Collectively, the omics-guided findings synthesized here suggest that structural complexity in plasmonic nanoarchitectures is not merely a synthetic achievement but a tunable determinant of cellular state, with important implications for the rational design of regenerative nanomedicines targeting neurodegenerative diseases and age-related neuronal decline.

## 1. Introduction

Neurodegenerative diseases and age-associated neuronal decline remain significant and unresolved medical challenges. These conditions are characterized by the progressive loss of neurons and dysfunction within the glial environment that typically supports neuronal health, particularly in the context of chronic oxidative stress, persistent neuroinflammation, and impaired cellular maintenance mechanisms [1,2]. Despite extensive research over several decades, current clinically approved treatments have failed to achieve sustained and broadly effective neuronal regeneration. This highlights the imperative for therapeutic approaches capable of actively reprogramming cellular states rather than merely decelerating disease progression.

Nanoparticle-based platforms have emerged as powerful tools for modulating cellular behavior because their size, shape, surface chemistry, and mechanical properties can be engineered with high precision [3,4]. These physicochemical parameters strongly influence how nanoparticles interact with biomolecules, enter cells through endocytic or phagocytic pathways, and engage intracellular signaling networks [5,6]. Beyond functioning as passive delivery vehicles, accumulating evidence indicates that engineered nanomaterials can directly modulate gene expression programs, epigenetic modifications, and redox homeostasis: processes intimately associated with neuronal survival and regeneration [7,8]. For example, lipid nanoparticles have been successfully implemented in clinical settings as mRNA delivery systems, whereas gold- and cerium oxide-based nanoparticles have demonstrated efficacy in preclinical studies for mitigating oxidative stress, attenuating neuroinflammatory signaling, and facilitating lineage conversion or neuroprotection [9,10].

Among engineered nanomaterials, gold nanoparticles have garnered significant attention owing to their tunable surface plasmon resonance, biocompatibility, and versatile surface functionalization [11,12]. Their biological effects are highly contingent upon their structural characteristics; parameters such as size, shape, surface charge, and internal architecture collectively influence cellular uptake, intracellular trafficking, and subsequent signaling pathways [13,14]. Concurrently, these plasmonic configurations are critical for biosensing applications, as increased structural complexity can enhance near-field confinement, increase hotspot density, and improve signal transduction efficiency in surface-enhanced Raman scattering (SERS) and localized surface plasmon resonance (LSPR) sensing platforms [15,16,17].

The architecture-dependent behavior observed in plasmonic nanostructures has prompted the development of highly intricate nanoarchitectures that extend beyond simple spherical or rod-shaped forms [18,19]. These advanced designs include internal porous networks, nested frame structures, branched topologies, and multi-compartment configurations. Such structural complexities enhance surface-to-volume ratios, generate dense electromagnetic hotspots, and establish biophysical interfaces that fundamentally differ from those of conventional nanoparticles. Despite increasing interest in these systems, the molecular mechanisms by which architectural complexity influences cellular aging and neuronal regeneration remain poorly understood. In particular, the field still lacks a sufficiently integrated, omics-guided synthesis of how nanoparticle architecture relates to transcriptomic programs and regenerative outcomes.

This review addresses that gap by integrating recent advances in the engineering of high-complexity plasmonic nanoparticles with published omics-guided evidence of their cellular effects. Unlike many previous reviews that primarily emphasize nanoparticles in terms of cargo delivery or material composition, this work considers architectural complexity itself as a modifiable factor influencing cellular state, transcriptomic responses, and plasmonic performance pertinent to biosensing applications. Initially, we organize the literature according to recurring structural motifs, including porous, frame-based, nested, branched, and multi-compartment architectures, and examine how these features relate to oxidative stress regulation, inflammatory signaling, neuronal lineage programs, and regenerative phenotypes. Throughout the review, nanoporous gold nanorods (AuNpRs) are used as a representative mechanistic case study because this platform combines a clearly definable structural parameter—internal porosity—with published transcriptomic datasets and functional regenerative outcomes [9]. Collectively, this perspective posits that nanoparticle architectural complexity represents not only a significant synthetic accomplishment but also a controllable means to modulate cellular states, with profound implications for regenerative nanomedicine, neurotheranostics, and age-related neuronal decline.

## 2. Architectural Complexity in Nanoparticles

### 2.1. Simple to High-Complexity Nanoparticle Architectures

**Figure 1:**
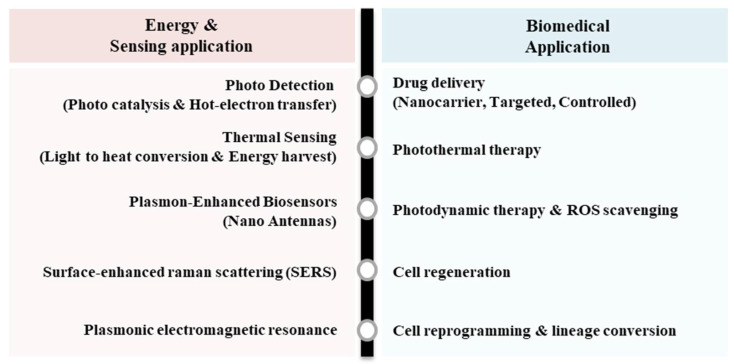
Schematic illustration of nanoparticle design and its application.

The field of nanoparticle engineering has undergone a paradigmatic shift from conventional spherical designs toward architecturally sophisticated platforms that demonstrate unprecedented structural complexity and multifunctional capabilities. High-complexity nanoparticles, characterized by intricate three-dimensional architectures such as porous networks, frame-type shells, nested “nanolens” constructs, branched multipods, and multi-compartment hybrids, have emerged as advanced platforms that surpass the limitations of traditional spherical nanocarriers [9,19,20,21,22,23,24,25,26,27,28]. These sophisticated architectures incorporate multiple functional domains within single nanostructures, enabling application ranging from plasmon-enhanced photodetection and SERS-based biosensing to drug delivery, and therapeutic interventions through precisely engineered geometries and surface modifications [29] (Figure 1).

To provide a clearer structural taxonomy for the remainder of this review, we qualitatively group representative plasmonic nanoarchitectures into five recurring categories—simple, porous, frame-based, nested-type, and multi-compartment systems—based on their overall morphology and field-confinement motifs. As summarized in Table 1, each category includes specific nanoparticle types (e.g., nanospheres, nanorods, nanoporous Au nanorods, Au–Ag nanoframes, Au nanolenses, N-th order nanoframes, octahedron-in-cube hybrids), along with their key structural features and typical plasmonic or functional properties. This qualitative framework is used in the following sections to relate structural motifs to biosensing performance and to biological outcomes in neural and retinal regeneration.

### 2.2. Nanoparticle-Based Therapeutics Platforms in Neurological Disorders

**Figure 2:**
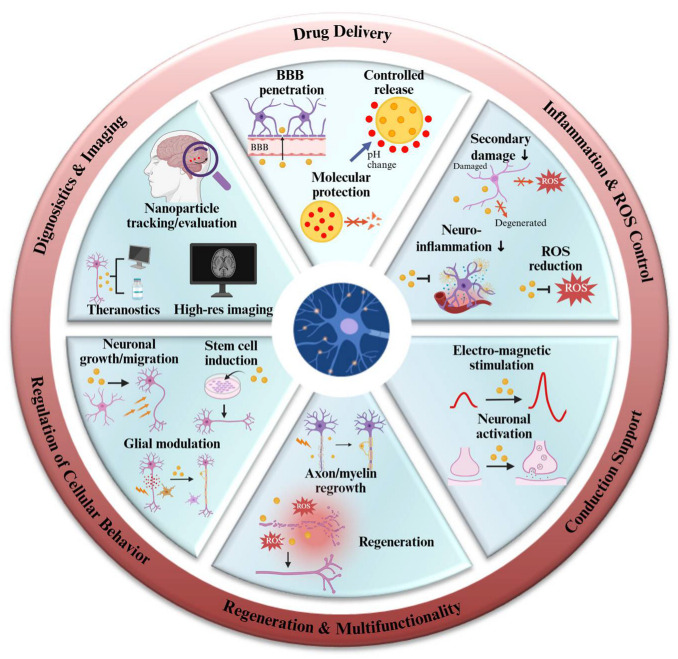
Nanoparticle-based therapeutic platforms and their functional contributions to neuronal repair including drug delivery, inflammation and ROS control, conduction support, regeneration, diagnostics, and regulation of cellular behavior. Created with BioRender.com.

To provide a functional framework for interpreting nanoparticle design complexity in neurobiology, we organized reported nanoparticle applications into six recurring operational modules (Figure 2): (i) drug delivery (including BBB penetration and controlled release), (ii) inflammation and ROS control, (iii) conduction support via external stimulation interfaces, (iv) regeneration-associated processes such as axonal outgrowth and remyelination, (v) regulation of cellular behavior including glial modulation and cell-state transitions, and (vi) diagnostics and imaging. This functional taxonomy highlights that, beyond carrier functions, many nanoparticle platforms are deployed to modulate cellular maintenance–related processes, particularly oxidative stress handling and neuroinflammatory tone, which are also tightly linked to aging-associated tissue vulnerability.

We next mapped representative nanoparticle systems across major neurological indications (Table 2) and found that distinct disease contexts converge on a limited set of biological endpoints. Across neurodegeneration (Alzheimer’s disease (AD), Parkinson’s disease (PD), Huntington’s disease and retinitis pigmentosa) [2,30,31,32,33,34,35,36,37] and injury models (spinal cord injury, stroke and traumatic brain injury) [38,39,40,41,42,43,44,45,46], nanoparticle approaches recurrently target (i) reduction in oxidative stress and lipid peroxidation, (ii) attenuation of innate immune and inflammatory signaling, and (iii) support of tissue remodeling programs such as angiogenesis, axon/myelin regrowth, or functional recovery.

For instance, in AD, lesion-targeting polymeric nanoparticles carrying small interfering RNAs (siRNAs) can be activated within high reactive oxygen species (ROS) microenvironments to downregulate BACE1 and caspase-3. This process reduces reactive astrocytes and improves memory performance in AD mouse models [30]. In PD models, elongated nanoporous Au facilitates the direct conversion of astrocytes into dopaminergic neurons by mitigating reprogramming-associated oxidative stress and upregulating antioxidant-related molecules, thereby enhancing motor behavior [9]. In Huntington’s disease, chitosan- or polymer-based nanoparticles have been employed to deliver siRNA or aggregation-blocking peptides via intranasal or systemic administration, resulting in decreased mutant huntingtin expression and alleviation of motor and synaptic deficits [35,37].

Nanoparticle platforms exhibit significant potential for the treatment of retinal degenerative diseases, including retinitis pigmentosa and age-related macular degeneration. Polymeric nanoparticles composed of poly(3-hexylthiophene-2,5-diyl) (P3HT) or neurotrophic factors have been demonstrated to restore physiological signaling at the cortical level and visually driven activities in rat models of retinitis pigmentosa [47,48]. Concurrently, plasmonic gold nanorods administered via intravitreal injection have been shown to function as injectable retinal prostheses [49]. Upon stimulation with near-infrared laser light, these nanoparticles activate bipolar and ganglion cells, thereby partially restoring light-evoked responses in models of retinal degeneration. Within this context, complex plasmonic architectures, such as porous nanorods, nanolenses, and nanoframes, are particularly promising for these applications. Their engineered hot-spot distributions and tunable near-infrared resonances can be optimized to align with ophthalmic stimulation windows, while simultaneously providing ROS-scavenging and anti-inflammatory effects.

At the nano-bio interface, the size (typically 1–100 nm), shape, and surface chemistry of nanoparticles critically influence their interactions with biomolecules, cellular internalization via endocytosis or phagocytosis, and subsequent trafficking to subcellular compartments. These physicochemical properties can be exploited not only for drug delivery but also to modulate synaptic neurochemistry. For example, pH-responsive nanoparticles have been engineered to enable controlled drug release and enhanced pharmacokinetics in models of depression [50]. Such smart formulations facilitate drug activation within neuroinflammatory or acidic brain regions, and their combination with psychotropic or antidepressant agents has been shown to enhance synaptic plasticity and catecholaminergic neurotransmission in neuropsychiatric disorders [51,52]. Conceptually, complex nanoparticles may function as nanocarriers that concentrate antidepressant molecules or modulate transporter activity (e.g., serotonin transporter, SERT) in the perisynaptic space, thereby providing an indirect mechanism to regulate serotonin reuptake and associated behavioral circuits.

Numerous nanoparticle-based platforms have been developed to target mitochondrial protection and redox regulation, which are essential for ATP-dependent functions and the stability of voltage-gated ion channels [53,54,55]. Mitochondria-targeted and ROS responsive nanoparticles, such as cerium oxide nanozymes and polymeric carriers, have been shown to mitigate oxidative damage, preserve mitochondrial ATP production, and improve functional outcomes in models of brain injury [53,54]. Furthermore, ATP-powered nanomotors (Apyrase@Au) have demonstrated that nanoparticle-mediated hydrolysis of endogenous ATP can generate localized protons, activate ion channels, induce Ca^2+^ influx, and direct neural stem cell differentiation in Parkinson’s disease models. These findings suggest that future designs of increased complexity could be tailored to simultaneously support ionic homeostasis, neurotransmitter balance, and regenerative remodeling.

### 2.3. Nanoparticle-Based Biosensing Platforms

High-complexity plasmonic nanostructures have been investigated not only for therapeutic modulation but also as signal-transducing components in biosensing applications. The architectural design critically influences electromagnetic field confinement and the robustness of signal readout. In plasmon-enhanced sensing techniques, including surface-enhanced Raman scattering (SERS) and localized surface plasmon resonance (LSPR) refractometric sensing, structural features such as intraparticle nanogaps, porous networks, and multi-rim/frame configurations enhance the density and uniformity of near-field “hot spots,” thereby improving signal-to-noise ratios and measurement reproducibility. This design principle is particularly pertinent to analytical performance, as SERS signal intensity is strongly dependent on local field enhancement; consequently, engineered near-field focusing can significantly amplify vibrational fingerprints, facilitating lower detection limits in diagnostic assays.

**Figure 3:**
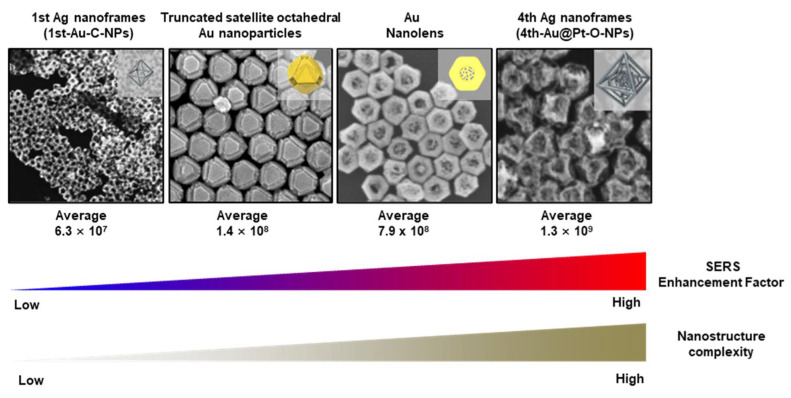
A conceptual summary of architectural complexity amplifies SERS signals. Adapted with permission from Ref. [15], 2026, Lee et al.

Representative evidence for complexity-driven signal amplification is demonstrated by plasmonic “nanolens” architectures, wherein a Pt@Au nanoring serves as a light-absorbing domain, and a central nanoporous Au network concentrates the electromagnetic field at the core. This configuration facilitates polarization-independent single-particle SERS with experimentally estimated enhancement factors averaging approximately 7.9 × 10^8^, demonstrating that internal architectural focusing can produce strong and reproducible SERS signals within individual particles [18,27] (Figure 3). Furthermore, multi-layered N-th nanoframes exemplify a scalable system in which increasing the number of internal frames and intraparticle coupling progressively amplifies single-particle SERS responses; reported enhancement factors rise from 6.3 × 10^7^ to 1.3 × 10^9^ as nested frame complexity increases, supporting the concept that “complexity” can serve as an engineering knob for sensing performance [28] (Figure 3). Similarly, particle-in-a-frame architectures, such as truncated satellite octahedral Au nanoparticles positioned along the edges of an outer octahedral structure, generate intraparticle nanogaps and enhanced near-field focusing, resulting in a heightened concentration of hot spots that enable sensitive SERS detection of small-molecule analytes [15] (Figure 3). Collectively, these findings provide a mechanistic basis for integrating complexity metrics into biosensor design: complex plasmonic structures function as intrinsic electromagnetic amplifiers, thereby supporting both diagnostic sensing (e.g., ultrasensitive molecular detection) and therapeutic monitoring in neurodegenerative and regenerative applications [18,56].

However, the relationship between architecture complexity and SERS performance must be interpreted with appropriate caution regarding reproducibility and generalizability. The biosensing relevance of architectural complexity should also be discussed with respect to reproducibility. High-complexity plasmonic nanoarchitectures can increase hotspot density and near-field focusing, and thereby support higher SERS enhancement, but this effect is not automatically robust across batches or experimental settings. Recent studies emphasize that reproducible SERS performance requires not only strong plasmonic coupling, but also precise nanogap definition, structural homogeneity, and controlled analyte access to hotspots [15,17,19,25,57,58]. Consistent with this view, recent complex nanoframe studies have highlighted highly homogeneous size and shape as prerequisites for reliable single-particle or ensemble SERS performance, rather than treating “complexity” alone as sufficient. We therefore use complexity as a comparative design parameter that can increase the potential for signal amplification, while explicitly recognizing that generalizability depends on batch fidelity, structural uniformity, and measurement context.

The development of systematic chemical toolkits has significantly advanced the synthesis of high-complexity nanoparticles, enabling the step-by-step construction of specific architectures with nanoscale accuracy. Park et al. have shown that rational design and synthesis of frame-structured nanoparticles can be achieved using combinatorial chemical approaches, which allow for extraordinary control over both structural complexity and functional integration (Figure S1) [18,19,25,59,60]. These frame-structured architectures offer several distinct advantages over conventional nanoparticles, including dramatically increased surface-to-volume ratios (up to 100-fold enhancement), enhanced near-field electromagnetic focusing capabilities, and the ability to incorporate multiple therapeutic modalities within spatially distinct compartments of the same nanostructure.

### 2.4. High-Complexity Nanoparticles Applications in Induced Neuronal Reprogramming

**Figure 4:**
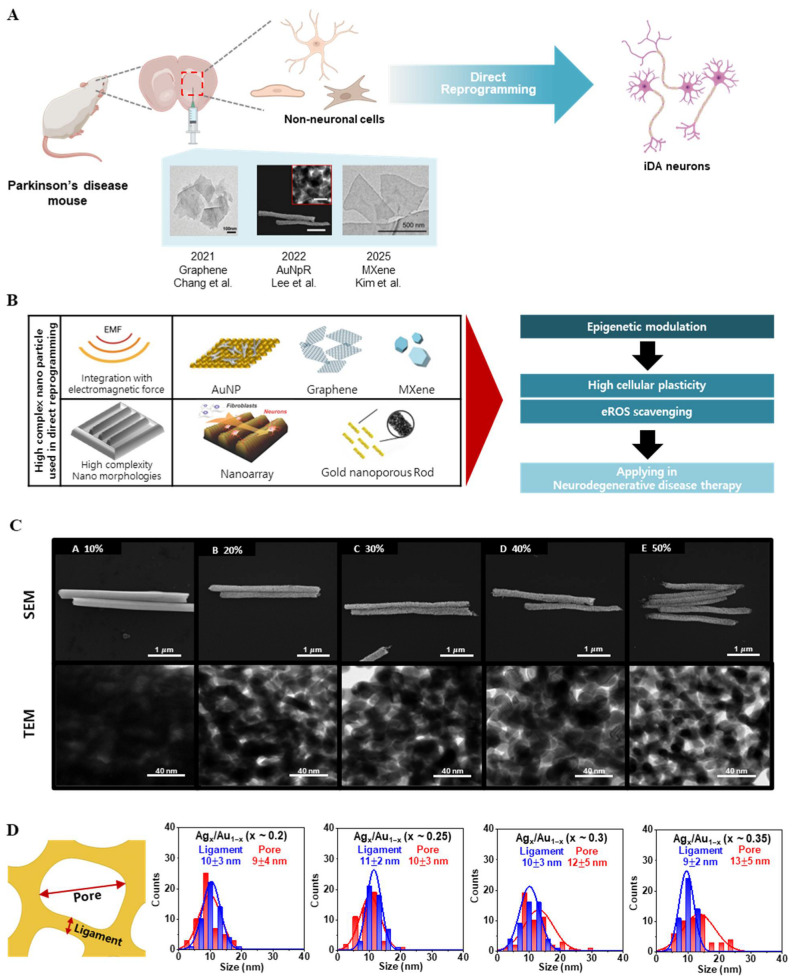
Representative effects and applicability of first-generation high-complexity nanoparticles in direct neuronal reprogramming. (**A**) Schematic overview of direct lineage reprogramming of non-neuronal cells into induced dopaminergic (iDA) neurons for Parkinson’s disease therapy. Previously reported first-generation high-complexity nanoparticle platforms—including nanoporous substrates, electromagnetized Au nanoparticles (AuNP), graphene nanoparticles, electromagnetized MXene, and elongated nanoporous Au networks—are shown with representative microstructural images. Created with BioRender.com. Adapted with permission from Ref. [62] 2021, Chang et al.; [9] 2022, Lee et al.; [63] 2025, Kim et al. (**B**) Conceptual framework outlining key physicochemical features of high-complexity nanostructures relevant to cellular reprogramming. Icon partially created by Biorender. Adapted with permission from Ref. [8] 2017, Yoo et al.; [9] 2022, Lee et al.; [63] 2025, Kim et al.; [64] 2022, Yang et al. (**C**) Structural characterization of SEM images and TEM micrographs for AuNpRs synthesized at increasing porosity ratios (10%, 20%, 30%, 40%, and 50%). Adapted with permission from [9] 2022, Lee et al. (**D**) Quantitative analysis of pore and ligament size distributions of AuNpRs across porosity conditions. Gaussian-fitted histograms show a stepwise enlargement of both pore diameters and ligament thicknesses, validating precise control of nanoarchitectural parameters. Adapted with permission from [9] 2022, Lee et al.

The enhanced performance characteristics of high-complexity nanoparticles have been systematically validated through comprehensive biomedical evaluations. Over the past decade, multiple studies have shown that specifically engineered nanomaterials can facilitate the direct conversion of non-neuronal cells (such as fibroblasts or astrocytes) into induced neurons (Figure 4A) [7,8,9,61,62,63]. For example, applying external physical stimuli in tandem with nanomaterials has significantly improved reprogramming outcomes (Figure 4B). Magnetically responsive gold nanoparticles exposed to electromagnetic fields (EMF) have been used to reprogram fibroblasts into functional induced dopaminergic neurons with high efficiency, even demonstrating therapeutic potential in Parkinson’s disease animal models [62]. Similarly, nanoscale morphological cues (e.g., culturing cells on biophysical cue nanoarray) act as physical signaling stimulators and have been found to promote neuronal marker expression during reprogramming [64]. More recently, electrical/mechanical stimulation via triboelectric nanogenerators has been shown to accelerate the direct lineage conversion of fibroblasts into neurons, increasing conversion efficiency and yielding more mature neuronal phenotypes [65]. Notably, these nanomaterial-assisted reprogramming strategies have proven effective both in vitro (cell culture models) and in vivo (within living organisms), broadening their potential for clinical translation [65,66].

On a mechanistic level, high-complexity nanoparticles appear to modulate the cellular epigenetic landscape to enable neuronal reprogramming (Figure 4B). In particular, an increase in histone H3 lysine-27 acetylation (H3K27ac), a hallmark of open chromatin, has been identified as a key factor associated with induced neuronal conversion [8]. Elevated H3K27ac reflects a more permissive chromatin state that enhances cellular plasticity, thereby allowing non-neuronal cells to more readily express neuronal genes and adopt neuronal identity [8]. In addition, many of these nanomaterials confer bioactive benefits such as scavenging of reactive oxygen species (ROS), which helps to mitigate oxidative stress during the reprogramming process. For instance, cerium oxide-based nanocomposites can directly neutralize intracellular ROS, creating a more protective environment for cells undergoing fate change [9]. The combination of a favorable epigenetic state (e.g., H3K27ac-mediated open chromatin) and reduced oxidative stress (via nanoparticle-assisted ROS scavenging) produces a cellular environment highly conducive to successful induced neuronal reprogramming in both in vitro and in vivo settings [7,9,67].

One notable example is provided by Lee et al. (2022) [9], who demonstrated that nanoporous gold nanorods (AuNpRs)—a plasmonic high-complexity architecture with a tunable pore-ligament network—can significantly enhance direct somatic cell conversion (Figure 4C). Unlike spherical AuNP or smooth gold nanorods, AuNpRs are characterized by defined internal void fraction (approximately 10–50%). In that study, AuNpRs treatment markedly improved the efficiency of converting fibroblasts into induced dopaminergic (iDA) neurons both in vitro and in vivo. Relative to smooth Au nanorods or gold nanotubes, AuNpRs produced stronger induction of dopaminergic reprogramming markers, including TH, DAT, VMAT2, FOXA2, and GIRK2. Moreover, the porosity of these nanorods was systematically tuned by adjusting pore size and ligament thickness, and conversion efficiency rose monotonically with increasing void fraction (Figure 4D), establishing a representative high-porosity plasmonic architecture between architectural complexity and reprogramming outcomes.

Beyond conversion efficiency, AuNpR-treated cells enhanced neuronal maturation features, including thicker neurites and improved electrophysiological properties. In a 6-hydroxydopamine Parkinsonian model, AuNpRs administration increased dopaminergic marker expression, dopamine release, and behavioral recovery relative to control nanoparticle conditions. These findings indicate that internal porous architecture is not merely a structural variation but a determinant of reprogramming efficiency and functional neuronal outcome.

The mechanistic value of this platform is strengthened by its associated transcriptomic dataset. RNA sequencing data deposited under ENA accession PRJEB44107 showed that AuNpR treatment enriched both neuronal and antioxidant-related gene programs, including upregulation of Gsta4, Sirt3, Sod1, Sod2, Acox1, Mt3, and Cox6b2. CellNet and gene set enrichment analyses further demonstrated positive enrichment of dopaminergic-neuron and antioxidant signatures, together with reciprocal negative enrichment of fibroblast-associated programs, indicating that AuNpR treatment shifts cells toward a more reprogramming-permissive and pro-regenerative transcriptional state. This interpretation is supported by functional perturbation experiments: pharmacologic depletion of glutathione using ethacrynic acid, as well as shRNA-mediated suppression of Gsta4, attenuated the AuNpR-enhanced conversion effect, supporting oxidative stress buffering as one major functional axis linking architectural complexity to neuronal reprogramming efficacy.

It should be noted that systematic omics studies that explicitly parameterize nanoparticle complexity across multiple architecture classes remain limited. AuNpRs are therefore used here not as a proxy for all high-complexity nanoparticles, but as a mechanistically informative plasmonic case study in which a tunable complexity parameter can be directly linked to both transcriptomic and regenerative readouts.

## 3. Omics-Guided Mechanistic Insights into Nanoparticle-Induced Neural Regeneration

### 3.1. Why Omics Is Necessary to Interpret Nanoparticle-Mediated Neural Regeneration

Phenotypic outcomes such as cell survival, neurite outgrowth, marker expression, and enhanced behavioral performance serve as critical indicators in the field of regenerative nanomedicine. Nevertheless, these outcomes alone do not elucidate the mechanisms by which engineered nanoparticles modulate cellular states. In the context of neural regeneration, exposure to nanoparticles can affect several interrelated processes, including the regulation of oxidative stress, inflammatory signaling pathways, chromatin accessibility, alterations in cell fate plasticity, and neuronal maturation [8,9,68,69,70,71,72]. Given that these processes are regulated at multiple levels, omics-based approaches are particularly valuable for discerning whether a nanomaterial primarily functions by maintaining redox homeostasis, modulating immune or glial responses, altering transcriptional identity, or adjusting developmental programs.

Among the various methodologies, bulk transcriptomics has been predominantly employed in the extant literature, especially in investigations centered on neuronal reprogramming, neuroprotection, or tissue repair subsequent to nanoparticle exposure. Bulk RNA sequencing is particularly efficacious in detecting pathway-level alterations, such as the upregulation of antioxidant genes, enhanced expression of neuronal lineage programs, or diminished activity of inflammatory pathways. Although single-cell transcriptomics is utilized less frequently, it offers the advantage of discerning whether nanoparticle treatment primarily modifies the proportions of specific cell populations or induces state changes within a particular lineage. Additionally, other approaches, including epigenomic profiling, can elucidate whether nanoparticle-induced effects are associated with modifications in chromatin accessibility or permissive histone marks that facilitate lineage switching. Collectively, omics technologies provide a critical mechanistic framework that links nanoparticle design to regenerative outcomes, thereby enabling biologically meaningful interpretation.

In this review, omics is not considered a standalone discovery approach; rather, it is regarded as a mechanistic bridge connecting structural engineering and regenerative biology. The studies summarized in Table 3 demonstrate that transcriptomic and related omics data are most informative when interpreted in conjunction with well-characterized nanoparticle features, including porosity, internal compartmentalization, plasmonic nanogaps, and stimulus responsiveness. This integrative analysis is particularly critical for highly complex designs, where multiple structural variables may simultaneously change, and reliance solely on phenotype-based assays could lead to unwarranted mechanistic conclusions.

### 3.2. Transcriptomic Evidence from Nanoparticle-Assisted Neuronal Reprogramming

Among the currently available examples, AuNpRs represent a prominent case in which a tunable structural characteristic has been systematically investigated in relation to transcriptomic and regenerative outcomes [9]. AuNpRs are elongated porous rods fabricated via template-assisted electrochemical deposition followed by selective dealloying. This methodology enables precise modulation of internal porosity while maintaining the overall rod morphology. Consequently, AuNpRs are particularly valuable for mechanistic studies, as porosity can be intentionally manipulated as a structural variable rather than occurring incidentally. Both in vitro and in vivo studies have demonstrated that these nanorods facilitate the direct reprogramming of fibroblasts into induced dopaminergic neurons without necessitating an intermediate pluripotent stage. Moreover, they promote neuronal maturation and functional integration. Transcriptomic profiling within this system demonstrated that AuNpR treatment is associated with the activation of gene programs related to both neuronal lineage specification and antioxidant responses. Notable genes involved include Gsta4, Sirt3, Sod1, Sod2, Acox1, Mt3, and Cox6b2, alongside markers indicative of dopaminergic and neuronal identity. Enrichment analyses reported in prior studies further corroborate a positive association with dopaminergic neuron and antioxidant signatures, accompanied by a concomitant reduction in fibroblast-associated transcriptional programs. Collectively, these results indicate that AuNpR treatment extends beyond merely increasing marker expression in a limited context; rather, it appears to induce a comprehensive shift in the transcriptional landscape that favors neuronal conversion and survival.

Equally important, the AuNpR system provides limited causal evidence supporting this interpretation. Pharmacological depletion of glutathione using ethacrynic acid diminished the pro-conversion effects of AuNpRs, and knockdown of Gsta4 similarly attenuated the reprogramming-associated benefits. These findings suggest that oxidative stress buffering constitutes a functional pathway linking the porous architectural feature to neuronal conversion efficiency. Thus, AuNpRs serve as a mechanistically informative example of how a high-complexity plasmonic structure can actively engage molecular programs directly relevant to reprogramming and regeneration, rather than merely acting as passive scaffold or carrier.

### 3.3. Convergent Molecular Themes Across Nanoparticle-Enable Neural Repair

Although the number of omics-enabled studies in this domain remains relatively limited, the representative examples presented in Table 3 reveal several recurring mechanistic patterns. The predominant theme is redox regulation and antioxidant support. Across various nanoparticle platforms, neural repair or protection is frequently associated with gene expression changes involved in mitigating oxidative stress, regulating glutathione levels, supporting mitochondrial function, and enhancing detoxification processes. This pattern is particularly evident in the AuNpR example; however, similar mechanisms are observed in other neuroprotective nanomaterial systems, where the reduction in ROS correlates with the preservation of neuronal identity and survival. Given that oxidative stress significantly impairs both neuronal survival and lineage reprogramming, this convergence suggests that one of the principal biological functions of these complex nanoarchitectures is to modulate the intracellular redox balance toward a state conducive to neural repair.

A second recurrent theme pertains to the modulation of inflammation and glial cell states. In models of injury and neurodegeneration, transcriptomic alterations induced by nanoparticles often demonstrate decreased activity within innate immune or inflammatory pathways, accompanied by molecular signals indicative of a more supportive microenvironment. Depending on the specific context, these changes may reflect alterations in microglial activation states or a broader suppression of inflammatory gene expression programs, alongside enhanced reparative signaling in adjacent cells. Regardless of the particular cell types involved, the prevailing evidence in the literature suggests that nanoparticle-mediated neural repair is seldom exclusively neuron-autonomous; rather, the modulation of the local inflammatory milieu constitutes a critical component of the overall regenerative response.

A third prominent theme is the activation of neuronal development and cellular plasticity programs. Numerous studies have reported an enrichment of pathways associated with nervous system development, neuronal differentiation, neurite formation, synaptic maturation, and brain development following nanoparticle treatment. In the context of direct reprogramming, these developmental and plasticity signals coincide with the downregulation of donor cell identity markers, reflecting a transition toward a more flexible, lineage-permissive state. This aspect is particularly critical for neuronal conversion, where successful reprogramming requires not only the activation of neuronal target markers but also the coordinated repression of the original fibroblast program and the stabilization of the newly established neuronal gene expression profile.

Collectively, these findings indicate that nanoparticle-mediated neural regeneration predominantly involves three principal processes: modulation of oxidative stress, regulation of inflammatory or glial states, and activation of neuronal repair or developmental pathways. In other words, current omics data do not support the existence of a singular universal pathway through which all nanoparticles facilitate regeneration. Rather, the extant literature suggests that various nanoparticle platforms consistently engage a limited set of biologically significant mechanisms that are critically relevant to neural repair.

### 3.4. Current Limitations of the Omics Literature in Nanoparticle-Mediated Neural Regeneration

Despite these promising developments, current omics research exhibits several significant limitations. Primarily, the majority of studies rely on single-platform case analyses, wherein omics measurements are conducted on individual nanoparticle systems rather than across systematically varied libraries of structures. Consequently, it remains challenging to ascertain whether observed transcriptomic alterations are attributable directly to structural complexity, material-specific chemical properties, or secondary factors such as dosage, cellular uptake efficiency, or stimulation methods.

Second, comparing results across different studies presents significant challenges due to variations in the species utilized, injury models employed, cell types analyzed, and sequencing methodologies implemented. For example, a transcriptomic profile obtained from reprogramming murine fibroblasts into neurons should not be directly equated with a bulk-tissue signature derived from the human cortex, even if both datasets indicate involvement of similar pathways, such as DNA repair or reduced inflammation. Consequently, such comparisons are most informative when they emphasize shared biological processes rather than presuming strict one-to-one biological equivalence.

Third, the existing literature remains predominantly focused on a relatively limited range of architectures, particularly gold-based plasmonic systems, alongside a smaller subset of inorganic platforms designed for neuroregeneration. Consequently, the field has yet to develop a comprehensive architecture-parameterized omics map. Such a map would systematically vary structural features—such as porosity, branching, frame number, nanogap spacing, and compartmental organization—and subsequently assess outcomes using standardized omics endpoints. Therefore, this review presents AuNpRs as a mechanistically informative example, rather than implying that a broadly generalized relationship among complexity, omics, and regeneration has been definitively established.

Finally, omics studies in this domain remain more effective at identifying associations at the pathway level than at establishing causal relationships. To move beyond purely correlational interpretations, future research should integrate architecture-controlled nanoparticle libraries with time-resolved transcriptomic analyses, cell type–specific resolution, perturbation experiments, and standardized metrics for structural quality. Such approaches will be essential for discerning which aspects of complexity are genuinely biologically significant, which are merely correlated with other properties, and which are most likely to be critical for translation into regenerative nanomedicine.

Collectively, these omics-guided studies suggest that nanoparticle-mediated neural repair is predominantly linked to the mitigation of oxidative stress, modulation of inflammatory states, and activation of neuronal developmental pathways. Furthermore, they underscore the necessity for more comprehensive, architecture-controlled datasets to establish generalizable design principles.

## 4. Discussion

### 4.1. From Structural Complexity to Functional Modulation

This review critically examines the role of plasmonic high-complexity nanoarchitectures in modulating biological functions beyond the conventional paradigm of nanoparticles serving solely as passive carriers. Historically, nanoparticle systems were primarily engineered for drug or gene delivery, with design parameters predominantly centered on particle size, surface charge, and colloidal stability. More recently, however, the advent of structurally intricate designs—such as nanoframes, nested frame-in-frame configurations, nanoporous networks, chiral structures, and core-in-frame hybrids—has expanded the design landscape by incorporating internal compartments, nanogaps, hierarchical organization, and multi-domain interfaces. These features not only introduce novel physicochemical properties but also present new opportunities for influencing biological systems.

To facilitate discussion across this diverse landscape, this review organizes plasmonic nanoarchitectures according to recurring structural motifs, including porous, frame-based, nested, branched, and multi-compartment systems. This qualitative architectural organization makes it possible to compare how specific motifs influence near-field focusing, accessible interfacial area, and nano–bio interactions. Within this context, architectures exemplified by highly porous gold nanorods, nanolenses, and particle-in-frame designs occupy the upper range of structural intricacy because they combine pronounced anisotropy with dense distributions of plasmonically active nanoscale features. Importantly, the significance of such complexity extends beyond fabrication. By enhancing local curvature, internal surface area, nanogap density, and electromagnetic field confinement, high-complexity plasmonic structures create a distinct physicochemical environment at the nano–bio interface. These attributes may modulate cellular behavior through multiple, overlapping mechanisms, including oxidative stress regulation, altered protein adsorption and intracellular transport, amplification of local signal transduction, and modulation of transcriptionally permissive states.

### 4.2. Mechanistic Insights from Omics-Guided Evidence Synthesis

A central message of this review is that the structural complexity of nanoparticles can be linked to molecular programs that are interpretable through omics-guided evidence synthesis. Among the currently available examples, AuNpRs represent one of the clearest cases in which a controllable structural feature, specifically internal porosity, has been systematically evaluated alongside functional outcomes and transcriptomic profiles. In both published in vitro and in vivo studies, AuNpRs enhanced the direct conversion of fibroblasts into induced dopaminergic neurons without requiring a pluripotent intermediate [9]. These effects were associated with reduced oxidative stress and increased expression of antioxidant-related genes, including Gsta4, Sirt3, Sod1, Sod2, Acox1, Mt3, and Cox6b2. Furthermore, perturbation of the system via ethacrynic acid treatment or suppression of Gsta4 attenuated the pro-conversion effect, supporting oxidative stress buffering as a mechanistic pathway through which this porous architecture mediates biological activity.

Beyond the AuNpR case, the representative omics studies summarized in Table 3 indicate that nanoparticle-mediated neural repair most consistently converges on three biological themes. The first is redox regulation and antioxidant support, including pathways related to glutathione metabolism, mitochondrial resilience, and ROS detoxification [9]. The second is inflammatory and glial-state modulation, in which nanoparticle exposure is associated with attenuation of innate immune pathways or establishment of a more permissive local support environment [70,72]. The third is activation of neuronal development and repair-associated programs, including pathways linked to neurite extension, neuronal differentiation, and lineage-associated transcriptional states [68,69,71]. These recurring patterns suggest that omics technologies are not merely cataloging transcriptional changes after nanoparticle exposure, but are beginning to reveal a limited set of mechanistic axes through which structurally complex plasmonic systems may influence neural regeneration.

Collectively, the evidence presented herein supports a working model in which architectural complexity influences regenerative potential through a combination of redox modulation, permissive transcriptional states, and lineage-supporting gene programs. At present, however, this model is substantiated primarily by a limited number of plasmonic architectures, particularly AuNpRs, and should therefore be regarded as a mechanistically grounded yet still evolving framework rather than as a fully generalized principle.

### 4.3. High-Complexity Nanoparticles as Tools to Modulate Aging-Associated Cellular Programs

The intersection between complex nanoparticle responses and molecular programs relevant to neural maintenance has important implications for the study of cellular aging. As neural tissues age, they accumulate DNA damage, undergo progressive epigenetic modifications, exhibit chronic inflammatory activation, develop mitochondrial dysfunction, and experience a decline in proteostasis regulation. Many of these alterations can be identified through transcriptomic analyses. The evidence discussed in this review suggests that structurally complex plasmonic nanoparticles, particularly porous and frame-based designs, may engage multiple aging-relevant pathways simultaneously.

In this context, nanoparticle complexity should be conceptualized not merely as a physical attribute but as a design parameter that can actively influence cellular behavior. Porous and nanogap-rich architectures have the potential to enhance antioxidant defenses and stress-buffering mechanisms, while simultaneously promoting transcriptional programs associated with DNA repair, developmental signaling, and neuronal plasticity. This concept holds particular significance for direct lineage reprogramming, wherein successful cellular transitions are constrained by oxidative stress, epigenetic barriers, and incomplete maturation into functional neurons. By mitigating the oxidative stress associated with reprogramming and fostering a more favorable molecular environment, high-complexity nanostructures may improve both the efficiency of cellular conversion and the quality of functional maturation.

More broadly, this framework suggests that future nanomaterials could be engineered not only to deliver therapeutic agents but also to modulate cellular maintenance mechanisms. For example, one could envision architectures optimized to support DNA repair pathways, improve mitochondrial redox balance, mitigate detrimental neuroinflammation or enhance neuronal differentiation and neural integration. In this sense, architectural complexity emerges as a tunable engineering parameter for shaping how neural tissue responses to aging, injury, and regenerative intervention.

### 4.4. Limitations and Translational Considerations

Several limitations should be acknowledged. First, the current evidence remains insufficiently comprehensive to support a universally applicable complexity–omics–regeneration principle. Mechanistic insights are still derived from a limited subset of plasmonic architectures, notably AuNpRs and select frame-based systems. Accordingly, the conclusions presented herein are most robustly applicable to plasmonic porous and frame architectures, rather than to all nanoparticles that may be described as structurally complex.

Second, the current omics literature is still dominated by single-platform case studies rather than architecture-parameterized comparisons across systematically varied nanoparticle libraries. As a result, available studies are more effective at identifying recurring pathway-level associations than at establishing broadly generalizable design rules. In addition, cross-study comparisons are inherently complicated by differences in species, disease models, target cell populations, and sequencing pipelines. Such comparisons are informative at the level of shared biological processes, but they do not by themselves establish causality or mechanistic equivalence.

Third, although findings from the AuNpR model provide promising indications that architectural modulation can influence regenerative outcomes, definitive causal attribution remains elusive. Variables including porosity, aspect ratio, surface area, nanogap density, surface chemistry, and dosage are interrelated, complicating efforts to discern whether observed molecular responses are primarily driven by geometric factors, increased accessible surface area, altered cellular uptake kinetics, or modifications in local redox dynamics. Addressing this issue will require matched materials, controlled dosing, and systematically varied architectural libraries designed specifically for mechanistic dissection.

Fourth, substantial translational challenges remain. Within the AuNpR model examined, no notable elevations in glial fibrillary acidic protein (Gfap) or ionized calcium-binding adapter molecule 1 (Iba1) were observed under the tested conditions, while intracellular reactive oxygen species (ROS) levels decreased and antioxidant pathways were upregulated. These findings suggest that the local biological response did not involve overt inflammatory exacerbation in this preclinical context. However, such observation should be interpreted as evidence of local tolerability within a defined experimental framework, rather than as a comprehensive safety validation. Broader translational applicability will require systematic evaluation of biodistribution, long-term persistence and clearance, complement activation, protein corona formation, chronic neuroimmune interactions, and unintended accumulation in off-target tissues. These factors are likely to be strongly influenced by nanoparticle size, surface chemistry, internal architecture, and administration route.

Fifth, analogous limitations apply to biosensing-related claims. Although high-complexity plasmonic structures can enhance hotspot density and near-field focusing— thereby increasing the potential for SERS or related optical transduction—such enhancement does not inherently guarantee robust or broadly generalizable performance. Batch-to-batch variability, nanogap reproducibility, and structural heterogeneity remain critical limitations, especially for hotspot-dependent detection modalities. Therefore, future biosensing studies should report not only peak enhancement factors but also metrics that capture signal uniformity, batch variance, and distribution-level reproducibility. In this sense, structural complexity should be interpreted as a factor that can increase the potential for plasmonic signal amplification, whereas robustness and generalizability remain independent empirical questions that require dedicated validation across batches and experimental conditions.

### 4.5. Future Directions and Concluding Perspectives

Progress in this field will likely necessitate the integration of precise materials engineering with biological assays capable of elucidating the underlying mechanisms. A critical strategy involves the development of systematic libraries in which parameters such as porosity, branching, frame number, nanogap spacing, and compositional asymmetry are varied independently and then evaluated using standardized multi-omics workflows. Such studies would enable the field to move beyond isolated case studies and toward predictive understanding of how structural motifs map onto specific cellular programs.

Another promising direction is the integration of computational plasmonics, high-throughput synthesis, and machine learning–driven optimization. Given the vast design space associated with highly complex nanoarchitectures, predictive methodologies may be particularly valuable for identifying structures most likely to elicit desired biological outcomes, such as enhanced antioxidant capacity, reduced innate immune activation, or cell-state alterations conducive to neuronal reprogramming, prior to conducting extensive experimental screenings.

In summary, the literature synthesized in this review suggests that architecturally complex plasmonic nanoparticles should not be regarded merely as elaborated versions of conventional drug delivery systems. Rather, they can function as active modulators of cellular states, capable of altering redox homeostasis, influencing transcriptional programs, and affecting regenerative capacity. Although the field remains at an early stage and further mechanistic and translational research is necessary, the increasing convergence of nanostructural complexity with omics-based molecular responses and phenotypes associated with neural repair provides compelling justification for the continued development of sophisticated plasmonic architectures in regenerative bioengineering and aging-related interventions.

## Figures and Tables

**Table 1:** Qualitative structural categories of nanoarchitectures.

Structural Category	Nanoparticle Types	Structural Features	Typical Properties	Ref.
Simple	Au nanosphere	Isotropic, no internal voids; smooth surface	Single dominant dipolar LSPR	[20]
	Au Nanoshell	Core shell geometry without additional internal porosity	Tunable by core/shell thickness ratio	[21]
	Au Nanorod	Mild anisotropy with a defined longitudinal axis	Aspect-ratio-tunable resonance	[22]
Porous	Nanoporous Au Nanorod	Elongated rods with internal pore–ligament network	Distributed hot spots, enhanced SERS	[9]
	Nanoporous Au films	Bicontinuous pore–ligament morphology over extended areas	Large-area hot-spot networks, neural interfaces or scaffolds	[23]
Frame-based	Au–Ag nanoframes	Hollow frame-like edges with open corners	Edge-localized fields; multiple LSPR bands arising from frame geometry	[24]
	Pt–Au nanoframes	Bimetallic hollow frames with sharp ridges	Strong edge/interface coupling; tunable multispectral response	[25]
Branched	Au hexapods	Six protruding arms Strong 3D symmetry breaking	Intense tip hot spots; directional field localization	[26]
Nested	Au nanolenses	Ring–core or ring–network configuration forming an internal focusing cavity	Near-field focusing to a central hot zone; very high single-particle SERS enhancement	[27]
Multi-compartment	N-th nanoframes	Multi-layered frame-in-frame (“Nth” order) structures	Cascaded plasmon interactions; stepwise SERS enhancement with increasing frame number	[28]
	Octahedron-in-cube hybrids	Octahedral core nested within a cubic outer frame	Strong hybrid gap modes between core and frame; concentrated 3D hot spots	[19]

**Table 2:** Representative nanoparticle systems applied in neurological disorders, categorized by disease type, nanotechnology platform, and key expected biological outcomes. Upward arrows (↑) indicate an increase or improvement, and downward arrows (↓) indicate a decrease or reduction in the corresponding parameter.

Disease	Nanotechnology	Reported Outcomes	Ref.
Alzheimer’s Disease	ROS-responsive siRNA NPs	↓ BACE1/caspase-3, nerve protection	[30]
	CBD–nanochitosan	↓ Aβ plaques, ↑ learning/memory	[31]
	mAb–liposomes	↑ BBB permeability, ↓ cognitive decline	[32]
Parkinson’s Disease	Fe_3_O_4_ NPs	gut microbiota–disease link study	[33]
	Mito-targeted NPs	↑ mitochondrial function, ↓ oxidative stress	[2]
	AuNPs	motor recovery, oxidative stress normalization	[34]
Huntington’s Disease	Chitosan–siRNA NPs	↓ HTT mRNA, ↑ motor & neuropathology	[35]
	PLGA–peptide NPs	↓ polyQ aggregation, ↑ motor ability	[36]
	Cholesterol–NPs	↓ motor/cognitive deficits, restore cholesterol balance	[37]
Spinal Cord Injury	Flavonoid NPs/hydrogel	anti-inflammatory/apoptotic, functional recovery	[38]
	VPA–chitosan NPs	↓ IL-1β/IL-6/TNF-α, ↑ functional & histological recovery	[39]
	DC-Chol–PLGA VEGF NPs	angiogenesis, tissue regeneration, motor recovery	[40]
Stroke	CNPs	↓ ROS, ↓ infarct size	[41]
	ROS–GA NPs	↓ HMGB1 release, ↓ oxidative stress	[42]
	PLGA–rtPA NPs	↑ thrombus targeting, faster lysis	[43]
Traumatic Brain Injury	ROS–Curcumin NPs	↓ edema, ↓ oxidative stress	[44]
	Resveratrol NPs	↓ NLRP3/caspase-1, ↓ IL-1β/IL-18	[45]
	CeO_2_ NPs	↓ lipid peroxides, ↓ inflammation	[46]
Retinitis Pigmentosa	P3HT/NTF NPs	↑ visually driven activity, ↑ cortical responses	[47,48]
	Plasmonic gold nanorods	↑ bipolar/ganglion cell activation, ↑ light-evoked responses	[49]

**Table 3:** Nanoparticle platforms with transcriptomic signatures linked to neuroprotection and regeneration. Upward arrows (↑) indicate an increase or improvement, and downward arrows (↓) indicate a decrease or reduction in the corresponding parameter.

Nanoparticle Platform	Model	Omics Modality	Key Pathways	Regenerative Implication	Ref.
Nanoporous gold nanorods (AuNpRs)	Fibroblast to induced DA neuron conversion; 6-OHDA Parkinsonian mouse model	RNA-seq	↑ Antioxidant and neuronal genes (Gsta4, Sirt3, Sod1/2, Acox1, Mt3, Cox6b2)	Supports direct neuronal reprogramming by reducing oxidative stress and promoting a reprogramming-permissive state	[9]
Electromagnetized gold nanoparticles	Fibroblast to induced DA neuron conversion; 6-OHDA Parkinsonian mouse model	RNA-seq	↑ H3K27ac and pro-neuronal chromatin remodeling	Supports direct neuronal reprogramming by activating H3K27Ac histone modification promoting a induced Dopaminergic neuronal conversion	[8]
Electromagnetized gold nanoparticles	Adult hippocampal neurogenesis in the aged brain	Single-cell RNA-seq	↑ Neural stem cell and progenitor populations ↑ Activation of Kat2a-associated histone acetylation (H3K9ac)	Suggests nanoparticle-assisted stimulation can enhance age-compromised neurogenesis and cognition	[68]
Mg/Al layered double hydroxide nanoparticles	Neural stem cell differentiation Spinal cord injury model	RNA-seq	TGFBR2/TGF-β-related immunomodulatory signaling Anti-inflammatory and neurogenic transcriptional changes	Promotes endogenous neurogenesis and improves spinal cord repair through immune microenvironment modulation	[69]
MgFe-LDH/NT3 nanoparticles + ultrasound	Neural stem cell differentiation Spinal cord injury model	RNA-seq	Piezo1/NF-κB-linked neurogenic and anti-inflammatory pathways	Enhances neurogenesis and functional recovery after SCI	[70]
LDH nanoparticle-doped cellulose nanofiber scaffold (CNF-LDH-RS)	Transected SCI model	RNA-seq	RhoA/ROCK/Myosin II and neuroactive ligand–receptor pathways linked to axon growth orientation and circuit remodeling	Supports organized axonal regrowth and circuit reorganization	[71]
Neuron-like cell membrane-coated curcumin PLGA nanoparticles (MM-Cur-NPs)	Parkinson’s disease model	RNA-seq	↑ Enrichment of anti-inflammatory and anti-oxidant programs ↑ neuronal mitochondrial protein VDAC1 gene	Supports neuroprotection through inflammation reduction and apoptosis suppression in a PD context	[72]

## Data Availability

No new data were generated or analyzed in this study. Data sharing is not applicable to this article.

## Supplementary Materials

The following supporting information can be downloaded at: https://www.mdpi.com/article/10.3390/bios16050239/s1, Figure S1: Library of complex NP synthesis. This synthetic roadmap for complex nanoparticles starts with either triangular Au nanoplates or octahedral Au nanoparticles, and proceeds through steps such as selective Au etching, selective Pt deposition, well faceted Au growth, tandem reactions, eccentric Au growth, spiky Au growth, mild polyol Ag growth, and the Kirkendall effect. By choosing specific steps as needed, a variety of rim architectures with distinct nanogap geometries can be produced.
