# Supplementary material for: Omics-Guided Insights into Nanoparticle Complexity and Neural Regeneration
Source: Biosensors (Basel). 2026 Apr 24;16(5):239. doi: 10.3390/bios16050239 (PMC13204803; doi:10.3390/bios16050239)
Supplement: Supplementary file 1 [file biosensors-16-00239-s001.zip › biosensors-4233841-supplementary.pdf]

SUPPORTING INFORMATION

Omics-Guided Insights into Nanoparticle Complexity and Neural Regeneration

Yujung Chang [1,¶],Sungwoo Lee [2,¶], Garam Yang [1,3,¶], Seung Seon Yang [1], Min Park [4], Jessica Kim [1], Yoon Ha [5,*,] Sungho Park [2,*] and Junsang Yoo [1,*]

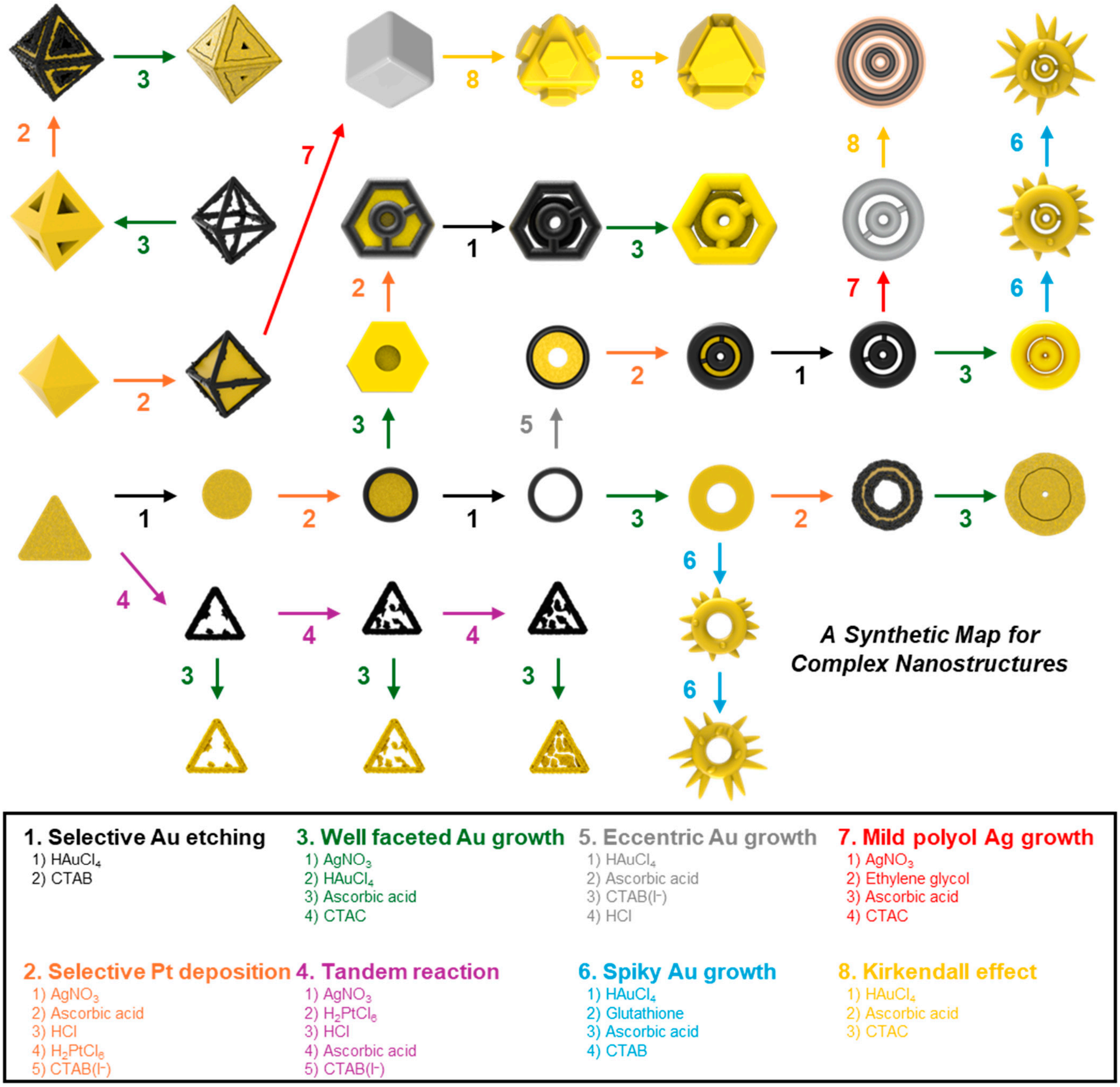

Figure S1. Library of complex NP synthesis. This synthetic roadmap for complex nanoparticles starts with either triangular Au nanoplates or octahedral Au nanoparticles, and proceeds through steps such as selective Au etching, selective Pt deposition, well faceted Au growth, tandem reactions, eccentric Au growth, spiky Au growth, mild polyol Ag growth, and the Kirkendall effect. By choosing specific steps as needed, a variety of rim architectures with distinct nanogap geometries can be produced.
